# The Impact of Three Different Canal Lubricants on Fracture, Deformity and Metal Slivering of ProTaper Rotary Instruments

**Published:** 2014-03-08

**Authors:** Yazdan Shantiaee, Omid Dianat, Farnoud Sharifi, Golnaz Nahvi, Golbarg Kolahi Ahari

**Affiliations:** a * Department of Endodontics, Dental School, Shahid Beheshti University of Medical Sciences, Tehran, Iran;*; b * Iranian Center for Endodontic Research, Research Institute of Dental sciences, Shahid Beheshti University of Medical Sciences, Tehran, Iran*; c * Endodontic Department, Dental School, Tehran University of Medical Sciences; *; d *Dental Student, Shahid Beheshti University of Medical Sciences, Tehran, Iran;*; e * Departmentof Biology (Biochemistry), School of Science, Payam e Noor university, Tehran, Iran*

**Keywords:** Canal Lubricants, EDTA, File Deformity, File Fracture, RC-Prep

## Abstract

**Introduction:** The aim of this *in vitro* study was to evaluate the effect(s) of three canal lubricants *i.e.* sodium hypochlorite, RC-Prep as the paste form of ethylenediaminetetraacetic acid (EDTA) and aqueous EDTA on the occurrence/incidence of fracture, deformity and metal slivering of ProTaper rotary instruments. **Methods:** A total of 120 mesial canals (*i.e.* mesiobuccal and mesiolingual) of first mandibular molars or buccal canals (*i.e.* mesiobuccal and distobuccal) of first maxillary molars, with curvatures of 10-20 degrees were selected and randomly divided into three groups of forty samples each. These selected canals all had approximate 19-21 mm working length and apical diameter equal to a #15 K-file. In each group, the root canals were prepared using ProTaper rotary instruments with an electric motor using one of the three aforementioned irrigants. Subsequently, samples were compared to each other at different magnifications (16×, 20×, 40× and 57×) for any fracture, deformity or metal slivering, by the Cox regression analysis. **Results**: The fractures rate of samples in RC-Prep group was significantly higher compared to other groups (*P*=0.01). No evidence of instrument deformity was detected in any groups. A statistically significant reverse relation between metal slivering and instrument fracture was observed. **Conclusions:** Application of aqueous EDTA and/or sodium hypochlorite as intracanal lubricants caused less fracture of ProTaper instruments compared to canal lubrication with RC-Prep.

## Introduction

Cyclic fatigue and excessive torsional stress have been identified as the main reasons for fracture of rotary nickel-titanium (NiTi) instruments. During root canal treatment, lubricants are mostly used to reduce frictional resistance between the rotating instruments and float debris produced after mechanical instrumentation. In addition, purported function of the chemical additives of the lubricant may alter the root canal dentin in a way that the mechanical action of endodontic hand or rotary files is facilitated [1, 2].

Calcium-chelating agents such as 17% ethylenediamine-tetraacetic acid (EDTA) can soften the root canal walls by affecting the nonorganic matrix of dentin (*i.e.* hydroxyapatite) and creating stable calcium complexes which is present in the dentin mud, smear layers or calcific deposits along the canal walls [3], whereas sodium hypochlorite (NaOCl) denatures the organic dentin matrix (*i.e. *collagen fibers) [4]. Consequently, both NaOCl and chelators can reduce the microhardness of dentin by acting on calcified tissues [5]. Furthermore, the impact of lubricant type on torsional load during rotary instrumentation appears to be significant. It is shown that a paste-type lubricant containing EDTA in a carbowax base [6], was not as beneficial as its aqueous counterpart when used in conjunction with rotary instruments (ProFile and ProTaper) in simulated root canals in human dentin [7]. Anyway, paste-like lubricants are more commonly suggested by manufacturers of rotary NiTi instruments during root canal preparation. Aqueous irrigators reduce torque, but the paste-type substances appear to be more effective in actively cutting files [8]. It is widely believed that lubrication during root canal preparation would decrease the mechanical stress on rotary instruments and therefore lowers the instrument separation [9]. However, not much information is available in the literature to approve or disapprove the best type of lubricant.

This claim has not been sustained yet in human teeth specimens. To date there is no information available about the incidence of fracture, distortion or metal slivering of the ProTaper rotary instruments when used with different types of aforementioned lubricants. Therefore, the aim of this *in vitro* study was to assess the impact of three different lubricants (NaOCl, RC-Prep and EDTA) on fracture, deformity and metal slivering of ProTaper instruments.

## Methods and Materials

In this *in vitro* experimental study, 120 mesial canals (mesiobuccal and mesiolingual) of extracted human first mandibular molars or buccal canals (mesiobuccal and distobuccal) of first maxillary molars were used. Immediately after extraction, all soft tissues and calculus were removed mechanically from the teeth and all the teeth were radiographed to verify the presence of mature apex and absence of any resorption or endodontic obturation.

The teeth were decontaminated by immersion in 5.25% NaOCl (Golrang, Pakshoo Co., Tehran, Iran) for 30 min and then they were stored in 0.9% sterile normal saline (Saman Co., Mashhad, Iran) at room temperature. The storage time of all teeth was less than 2 months before the initiation of experiment.

Root curvatures were determined using the Schneider’s method [10]. According to the category of Wein, all selected mesial canals of mandibular molars were type III [11]. Canals with 10 to 20 degrees of curvature were selected. All the teeth were mounted in acrylic resin (Khosh Resin Co., Tehran, Iran) and stabilized in a small vise grip. The access cavity was prepared using fissure bur (012, Tizkavan, Tehran, Iran) installed in a high speed handpiece and water coolant.

For each canal, the working length was determined by passing #10 K-Flexofile (Dentsply Maillefer, Ballaigues, Switzerland) and was between 19 and 21 mm from the anatomic foramen. The apical portion of canal was enlarged to a size #15 K-file (Dentsply Maillefer, Ballaigues, Switzerland). Then new ProTaper files were introduced to the canals. Before use, all files were visually inspected at 16×, 20×, 40× magnification using stereomicroscope (Olympus optical Co., LTD, model SZX-1 LLB200, Tokyo, Japan) to assure the absence of any distortion, metal slivering or deformity. In case of any distortion, the file was excluded.

The selected canals were randomly divided into one of three experimental groups of 40 teeth each (A, B, C).The samples were randomly assigned to one of three groups by a computer algorithm (Microsoft Excel, Excel version in Microsoft Office 2007 for Windows 7) that stratified treatment allocation by the curvature of the samples. To determine equal amounts of lubricants, 0.5 μL of each aqueous lubricants [*i.e.* group A; 1% NaOCl and group B; 17% ethylenediaminetetraacetic acid-EDTA (Asia Chemi Teb Co., Tehran, Iran) with pH=8] was placed in canals using a pipette with a disposable tip. For the group C, the canals were filled with RC-Prep lubricant paste (Premier Dental Products, Philadelphia, USA) between instruments.

In group A, after placing 0.5 μL of 1% NaOCl, ProTaper files (shaping and finishing files) were used in a pecking motion as follows: file S1 (#10/variable taper) was advanced to the resistance point which would not exceed more than two-thirds of the canal depth. Other files were used to the working length (19-21 mm), in the following sequence: S2 (#15/variable taper), F1 (#20/0.07), and F2 (#25/0.08) to achieve the apical size of 25. Each file was used to the full working length and rotated in that length for duration of 2 sec Before changing to the next file the canals were irrigated with 2 mL of normal saline.

In group B, 0.5 µL of 17% EDTA was placed in canals before each file insertion and rotary instrumentation technique was done the same as group A.

In group C, the canals were filled with RC-Prep, using a 26-gauge irrigating needle before each file insertion. The ProTaper files were used with the same protocol as group A.

The whole procedure was performed by one investigator. Instrumentation procedure was performed according to the manufacturer’s instruction. A particular principle was used for using the proper number of the files; each ProTaper instrument was limited to a maximum number of 10 canals. If the instrument showed fracture before that time, the data was recorded and the file was replaced with a new one. None of the files were forced into the canal. The instrumentation speed, torque, and force were controlled by a control unit, Endo IT professional (Aseptico Inc., Woodinville, WA, USA) [4].

**Figure 1:**
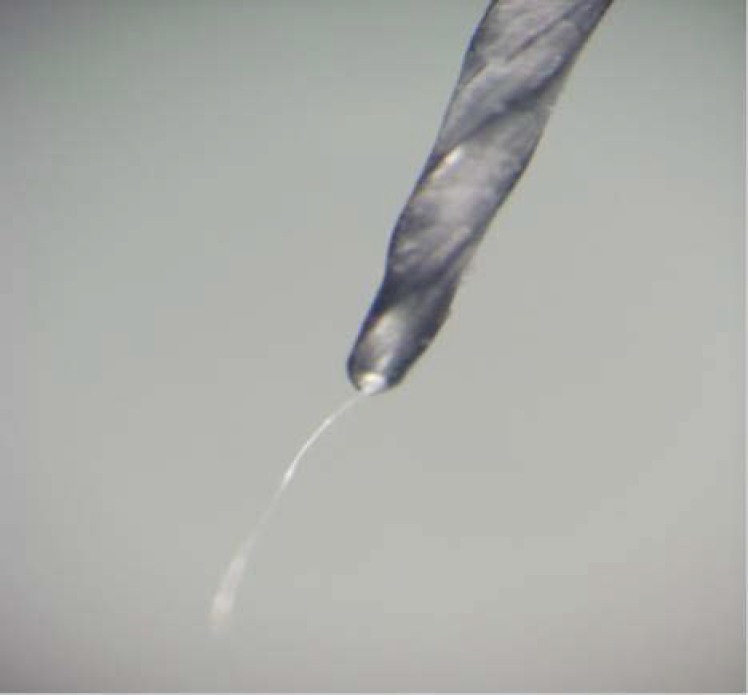
A stereomicroscopic view of wire slivering in F2 ProTaper file (20× magnification

After finishing preparation of a canal, the blade of, each file was divided into segments of 4 mm length and each segment was separately inspected in different directions for deformity, fracture or metal slivering under 16×, 20×, 40× and 57× magnifications. Generally, these instruments were discarded after evident permanent deformation, fracture or reaching to maximum limit as mentioned. Metal slivering was defined as a condition in which a thin layer of metal is separated from the outer surface of the cutting edge while it is attached to the distal end of the file (Figure 1).

Data were subjected to analysis using SPSS statistical software, version 12. All groups were compared to one another for any deformity, fracture or metal slivering by “Cox regression analysis”.

## Results

**Figure 2:**
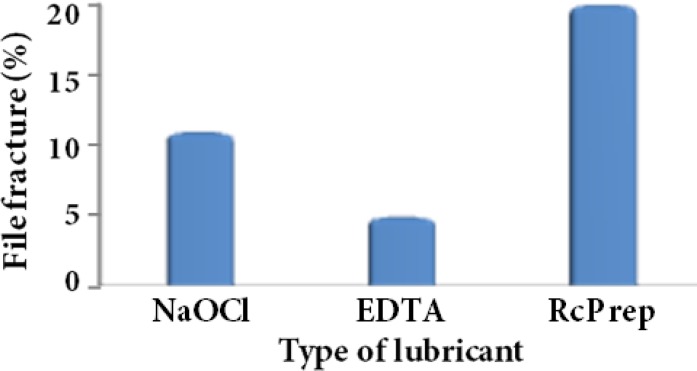
Diagram of fracture rate in ProTaper instruments (%)

The total number of files used for canal preparation was 18 files for group A [S1 (*n*=5), S2 (*n*=4), F1 (*n*=5), F2 (*n*=4)], 17 files for group B, [S1 (*n*=4), 4 S2 (*n*=4), F1 (*n*=5), F2 (*n*=4)] and 20 files for group C [S1 (*n*=5), S2 (*n*=5), F1 (*n*=5), F2 (*n*=5)] (Figure 2).

In this study, there was no evidence of deformity in instruments. There were 6 cases of metal slivering and 2 cases of instrument fracture in 1% NaOCl group. Seven cases of metal slivering and only 1 case of instrument fracture in aqueous 17% EDTA group were found, and 4 cases of metal slivering and 4 cases of instrument fracture in RC-Prep group were noted. There was a statistically significant difference in instrument fracture between RC-Prep group and other groups (*P*=0.01), but the difference between EDTA and NaOCl groups was not significant. Fracture rate was calculated between different groups and the least fracture rate was noted in EDTA group.

Also, a statistically significant reverse relationship between metal slivering and instrument fracture was observed.

## Discussion

The purpose of this *in vitro* research was to investigate the rate of deformity, breakage and metal slivering that may occur during the use of ProTaper rotary instruments with different lubricants (NaOCl, EDTA, RC-Prep) in root canals of extracted molars. Given the results, there was no evidence of instrument deformity in all three groups. The RC-Prep group showed more file fractures.

According to previous studies, one possible explanation for the different rates of file fracture in different groups may be related to different forms of lubricants [6, 8]. Because of paste-like form of RC-Prep, when mixed with dentin debris in canals, a thicker mud is formed that causes more friction with dentin walls, but in aqueous EDTA we did not observe the same result which can be attributed to the liquid form of this lubricant.

Even though NaOCl has a liquid form, the file fracture percentage was higher than EDTA group and that may be because of the less lubricative effect of NaOCl compared to EDTA. Another reason for this fact can be the chelating effect of EDTA. After using EDTA in canals, stable calcium complexes are formed in dentin mud, smear layers or calcified deposits, but not in normal dentin. Consequently exerted force on files will reduce and they tend to fracture less. However, the differences between two liquid lubricants were not statistically different.

All the teeth selected for this study were extracted less than two months before the study and stored in normal saline to simulate the clinical conditions of normal dentin but in many studies the exact extraction time of the teeth was not mentioned [6, 8].

The effect of liquid chelating solutions on instrument fracture or deformity has been predominantly reported in previous literatures, but no information is available regarding the effects of lubricants on metal slivering of rotary files. That may be because of not using the microscope for accurate assessment of instruments [12].

In this study, selected motion for canal instrumentation was pecking or pumping [13], but in some studies, type of file movement was not mentioned [6]. The current study was an attempt on simulating clinical conditions encountered during root canal preparation in a standardized environment. Nevertheless, the current study allowed us to investigate the reaction of different lubricants with dentin, a phenomenon that has received little attention so far, given that the majority of researches used plastic blocks to evaluate root canal instruments [7], that does not mirror a clinical situation because physical and chemical features of blocks differ from human dentin.

In this study, the instrumentation torque, speed and force was controlled simultaneously by Endo IT control unit, according to manufacturer’s protocol. But in many studies, all these factors were not simultaneously controlled [14, 15].

The torque-controlled motor used in the current study (Endo IT) was the same as the one used in a study by Weiger R *et al.* on FlexMaster and high speed rotary instruments [16]. Consequently all the factors influencing instrument fracture, deformity or metal slivering, except for the lubricant type, were similar in all three tested groups.

In the present study, any sign of metal slivering, deformity or fracture was recorded after inspecting used files macroscopically and microscopically under different magnifications. The files were used until they reached the maximum limit of canal instrumentation (10 canals for each file) or until they broke. But in some studies, a new file was used for every single canal preparation that may decrease the fracture rate and permanent deformity. This study revealed no sign of instrument deformity or cracking in any groups which is similar to the results of other researches on ProTaper systems, because ProTaper files tend to break without deformity or cracking [2, 17, 18]. The ProTaper files used in aqueous 17% EDTA had the most number of file metal slivering but the least number of file breakage. Reverse relationship between files metal slivering and fracture of a file appears to support the hypothesis that metal slivering is a phenomenon that suddenly removes excessive force on the files and therefore no fracture would happen in that area of the file because metal slivering would decrease stress in the mentioned area.

Using 1% NaOCl was less efficient than 17% EDTA under the conditions of this study. However, the concentration of this irrigant was relatively low in the present study. It needs to be taken into consideration that the NaOCl solution of higher concentration may have more effect on reducing the fracture and deformity of ProTaper rotary instruments. Previous studies showed that solutions with higher concentrations (>3%) substantially affect mechanical properties of dentin during root canal therapy, whereas lower concentrations did not [19]. More research is needed to identify which lubricant is beneficial in reduction of instrument fracture besides having no interference with disinfecting agents. This study presents only one brand of NiTi rotary file (ProTaper). More studies can be conducted to compare the effects of these lubricants on different current rotary instruments to make reasonable implication for general understanding.

## Conclusion

Under the limitation of this *in vitro* study, current observations confirmed that using RC-Prep as the canal lubricant during root canal preparation increases the fracture rate of ProTaper rotary instruments. It appears that lubrication of the canal system using aqueous solutions has greater benefit; *i.e.* reduction of the fracture risk in ProTaper rotary files.
